# Expression and Analysis of *TBX3* Gene in the Skin from Three Locations on Dun Mongolian Bider Horse

**DOI:** 10.3390/genes15121589

**Published:** 2024-12-11

**Authors:** Tana An, Manglai Dugarjaviin, Haige Han

**Affiliations:** Inner Mongolia Key Laboratory of Equine Science Research and Technology Innovation, Inner Mongolia Agricultural University, Hohhot 010018, China; 15848154479@163.com (T.A.); dmanglai@163.com (M.D.)

**Keywords:** *TBX3* gene, dun coat color, Mongolian horse, Bider marking

## Abstract

Background/Objectives: The Mongolian horse, one of the oldest and most genetically diverse breeds, exhibits a wide variety of coat colors and patterns, including both wild-type and unique features. A notable characteristic of dun Mongolian horses is the presence of Bider markings—symmetrical, black-mottled patterns observed on the shoulder blades. These markings are also seen in Przewalski’s horses. The dun coat color, a common wild-type phenotype in domestic horses, is characterized by pigment dilution with distinct dark areas and is regulated by mutations in the *TBX3* gene. This study aimed to investigate the role of *TBX3* in the development of Bider markings in dun Mongolian horses. Methods: Skin tissue samples were collected from three key anatomical regions of dun Mongolian horses with Bider markings: the croup, dorsal midline, and shoulder. Histological staining was conducted to examine the skin and hair follicle structure and pigment distribution. RT-qPCR was used to measure *TBX3* mRNA expression, while immunoblotting and immunohistochemistry were employed to analyze TBX3 protein levels and localization. Results: Hematoxylin and eosin staining revealed the skin and hair follicle structures, including the epidermis, hair shaft, and hair bulb across different stages of the hair growth cycle. Differences in pigmentation were observed across the sampling sites. The croup and the light-colored area of the shoulder showed asymmetrical pigmentation, while the dorsal midline and dark-colored area of the shoulder displayed symmetrical pigmentation. *TBX3* mRNA expression levels were significantly higher in the croup compared to the shoulder and dorsal midline; however, corresponding TBX3 protein expression did not show significant differences. Immunohistochemical analysis localized TBX3 protein predominantly in the hair bulb and epidermis. Conclusions: This study demonstrates region-specific differences in *TBX3* expression that correlate with pigmentation patterns in dun Mongolian Bider horses. These findings provide valuable insights into the molecular mechanisms underlying Bider markings, offering a deeper understanding of the genetic regulation of coat color and primitive markings in equines.

## 1. Introduction

The coat color of domestic horses has been shaped by both natural and artificial selection, resulting in a wide variety of phenotypes [1]. These variations have long been of interest to geneticists and evolutionary biologists [2]. Horses were domesticated approximately 4000 years ago [3], with the bay and black coat colors being the earliest identified [4]. Over time, selective breeding by humans has emphasized not only functionality and temperament but also aesthetically striking coat colors, which have been extensively studied at the genetic level [5]. Dun coat color is a wild-type trait characterized by a diluted body color and distinctive dark patterns known as primitive markings [6]. These markings include leg stripes, shoulder stripes/shading, dorsal stripes, and facial masks, which are rare in modern domestic horses but commonly found in indigenous and wild-type horses [6,7,8,9]. Among these primitive patterns, the Bider marking stands out as a unique, irregular black-mottled pattern observed exclusively on the shoulder blades of dun Mongolian horses and Przewalski’s horses [7,10]. In Mongolian culture, horses with Bider markings hold significant value and are often referred to by names such as “Flower Eagle Horse”, “Zagltai Horse”, and “Eagle Wings Horse” (Figure S1). These markings are not only visually distinctive but also carry cultural and historical importance, symbolizing traits such as strength and resilience. The formation of the dun coat color and its associated primitive markings has been linked to mutations in the *TBX3* gene [6,8]. While the genetic mechanisms behind the dun coloration are well documented, the specific pathways leading to the development of Bider markings remain largely unexplored. Given the consistent correlation between the dun phenotype and Bider markings in Mongolian horses, we hypothesize that *TBX3* expression plays a crucial role in the development of these distinctive shoulder patterns. This study aims to explore the role of *TBX3* in the formation of Bider markings in dun Mongolian horses. By analyzing *TBX3* expression at various anatomical sites, we seek to uncover how this gene influences asymmetrical pigmentation and contributes to the unique Bider pattern.

## 2. Materials and Methods

### 2.1. Sample Collection

**Figure 1:**
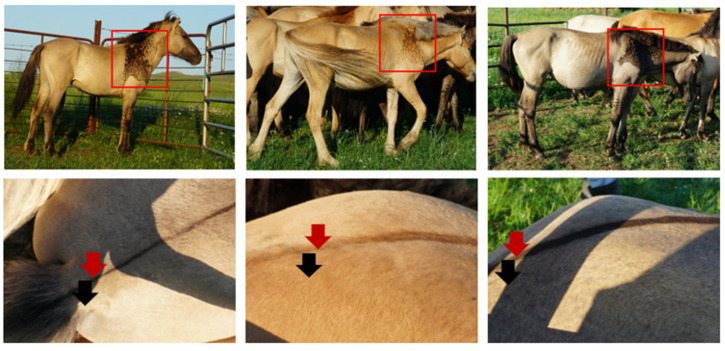
Sample collection from dun Mongolian Bider horses. **Left**–**Right**: Yellow dun, Red dun, Blue dun (Grullo). The corresponding Bider markings on the shoulder blade are indicated within the red boxes, the red arrows show the dorsal midline, and the black arrows show the croup skin.

Skin tissue samples were collected from dun Mongolian Bider horses (*n* = 3) at a farm in the East Wuzhumqin Banner, Xilingol League, Inner Mongolia Autonomous Region, in August 2022. Each sample, measuring 1 cm × 1 cm, was obtained from three distinct anatomical locations: the shoulder, dorsal midline, and croup (Figure 1). All surgical procedures were conducted under a combination anesthesia protocol, with careful measures implemented to minimize any discomfort experienced by the horses. Detomidine (0.01–0.02 mg/kg, IV) combined with butorphanol (0.02–0.04 mg/kg, IV) was used for analgesia. Samples were collected following the guidelines approved by the Animal Ethical Committee of Inner Mongolia Agricultural University. The shoulder area, where Bider markings appear, was further divided into light-colored and dark-colored regions. The dorsal midline was selected as it is a common primitive marking of dun horses, while the croup represented the diluted body color. The collected tissue samples were preserved using two methods. One half was quickly frozen in liquid nitrogen and stored at −80 °C for subsequent molecular analysis. The other half was fixed in 4% paraformaldehyde solution for use in sectioning, hematoxylin and eosin (HE) staining and immunohistochemical staining. After fixation, the samples underwent gradient dehydration and were stored at 4 °C for long-term preservation.

### 2.2. Paraffin Embedding and Sectioning

Fixed tissues were removed from the 4% paraformaldehyde solution (Solarbio Science & Technology Co., Ltd., Beijing, China) and sequentially dehydrated in ethanol concentrations of 75%, 85%, and 95% and twice in 100% ethanol to remove water while preserving tissue morphology. Dehydrated tissues were then treated with xylene in three 15 min cycles to achieve transparency. For wax infiltration, tissues were immersed in a soft wax–xylene mixture for 20 min, followed by full immersion in soft wax for 30–45 min under a continuous vacuum (0.05 MPa) to enhance wax penetration and eliminate air bubbles. They were then transitioned to a hard wax mixture for 20 min and deeply embedded in hard wax for up to 3 h under vacuum to ensure uniform coating and structural stability. The wax-embedded tissues were allowed to solidify for 24 h, wrapped in tinfoil to preserve shape and trimmed to expose the desired tissue region for sectioning. Using a microtome (Leica Microsystems, Wetzlar, Germany), wax blocks were sliced into ultrathin sections (5 µm thickness), unfolded on a 40 °C spreader to form flat sheets, and transferred to adhesion microscope slides (CITOTEST, Jiangsu, China). The slides were baked in a 65 °C oven for 2 h to improve adhesion and remove residual solvents, fully preparing them for subsequent staining and analysis.

### 2.3. Hematoxylin and Eosin (HE) Staining

HE staining was performed to visualize the skin tissue morphology using the sections prepared in Section 2.2. Before staining, the sections were examined under a microscope to ensure they were flat, free of air bubbles, and correctly oriented. The sections were then baked at 65 °C for 30 min to enhance adhesion to the slides and remove residual solvent. Dewaxing was conducted with two 10 min xylene treatments to ensure complete wax removal, followed by graded alcohol dehydration to rehydrate the tissues, with a 3 min dwell at each concentration. The sections were stained with hematoxylin solution for approximately 3 min to color the nuclei dark blue, rinsed briefly with tap water for up to 15 min to remove excess stain, and treated for about 20 s with a differentiation solution to eliminate over-stained hematoxylin. Two additional 5 min rinses with tap water followed to thoroughly remove the differentiation solution. The sections were then immersed in eosin staining solution for 30 s to stain cytoplasmic and mesenchymal components red or pink, creating a distinct color contrast. Post-staining, excess dye and water were removed through gradient alcohol dehydration, with a 2 min dwell at each step, followed by two xylene treatments for 5 min each to enhance tissue transparency. The stained sections were sealed with neutral tree glue, ensuring they remained flat and adhered to the slides. Once the glue dried naturally, the tissue morphology and hair follicle structures were visualized under a microscope (Olympus Sales & Service Co., Ltd., Tokyo, Japan).

### 2.4. RNA Extraction, Quality Testing, and cDNA Synthesis

Tissue samples were finely ground into a powder using the magnetic bead method. To prevent RNA degradation, all procedures were performed under frozen conditions. RNA was extracted following Invitrogen’s TRIzol method (Invitrogen, Carlsbad, CA, USA), yielding 30 µL of total RNA per sample. The purity and concentration of RNA were assessed using an enzyme marker, with acceptable purity indicated by a 260/280 ratio of 1.9 to 2.2 and integrity confirmed by RIN values between 6.7 and 7.3. The extracted RNA was subsequently converted into cDNA through a reverse transcription process. For the reverse transcription reaction, 2 µL of 5× PrimeScript RT Master Mix (Perfect Real Time) (TaKaRa Bio Inc., Dalian, China), which included random primers, was used. The RNA concentration was carefully adjusted to ensure the total RNA in the reaction system (10 µL) did not exceed 500 ng, optimizing reverse transcription efficiency. RNase-free dH_2_O was added to bring the total reaction volume to 10 µL. The reaction conditions consisted of an initial incubation at 37 °C for 15 min, followed by a brief hold at 85 °C for 5 s. The resulting cDNA was stored briefly at 4 °C before being transferred to a −20 °C freezer for long-term preservation.

### 2.5. Real-Time Fluorescence Quantitative PCR (RT-qPCR) for TBX3

Fluorescence quantitative PCR (RT-qPCR) technology was utilized to measure the expression levels of the *TBX3* gene, using *B2M* as the reference gene. Gene sequences were retrieved from NCBI, and primers were designed using Primer Premier 5.0 software. The β2-microglobulin (*B2M*), a housekeeping gene recognized for its stable expression across various cells and tissues, was used to compare the relative levels of *TBX3* mRNA, as described in a previous study [6]. The primers were synthesized by Shanghai Sangon Biotech, and primer sequences are given in Table S1. Following the TB Green Kit protocol, each sample was amplified in triplicate to ensure experimental reliability. The 25 µL reaction system comprised 12.5 µL of TB Green™ Premix Ex Taq™ II (TaKaRa Bio Inc., Dalian, China), 1 µL each of forward and reverse primers (10 µmol/µL), 2 µL of cDNA template, and 8.5 µL of dd H_2_O. Thermal cycling conditions included an initial pre-denaturation step at 95 °C for 30 s, followed by 40 cycles of denaturation at 95 °C for 5 s, annealing at 60 °C for 33 s, and extension at 72 °C for 30 s, conducted on a BIO-RAD CFX96™ Optics Module (BIO-RAD, Hercules, CA, USA). Post-reaction analysis provided the amplification curve, melting curve, and Ct values. Quantitative results were calculated using the 2^−∆∆CT^ method and analyzed using GraphPad Prism 10 software for visualization and statistical analysis. Before conducting the ANOVA and *t*-tests, we performed normality and homogeneity of variance tests on the data from *TBX3* gene expression across the shoulder, dorsal midline, and croup regions. The normality of the data was assessed using the Shapiro–Wilk test, and the results indicated that all data sets followed a normal distribution (*p*-values > 0.05). Additionally, Levene’s test was used to assess the homogeneity of variance across the groups, revealing no significant differences in variance (*p*-values > 0.05), confirming that the variances were consistent across the groups.

### 2.6. Extraction of Total Protein

Skin samples were removed from the −80 °C freezer and immediately placed into liquid nitrogen, where they were ground into a fine powder using a tissue homogenizer. To prevent degradation, the entire process was conducted under frozen conditions. The powdered samples were transferred to 1.5 mL centrifuge tubes, with 1 mL of lysate added for every 100 mg of tissue. Following the manufacturer’s instructions, a lysis buffer (Beyotime Biotechnology Co., Ltd., Shanghai, China) and protease inhibitor (Beyotime Biotechnology Co., Ltd., Shanghai, China) were mixed at a 99:1 ratio, and 1 mL of this mixture was thoroughly combined with the tissue powder for 1 min. All lysis steps were performed on ice or at 4 °C to maintain protein integrity. The tubes were then incubated on ice for 1 h to ensure complete lysis, followed by centrifugation at 12,000 rpm for 15 min at 4 °C. The resulting supernatant, containing the total protein, was carefully transferred to new centrifuge tubes and stored at −20 °C for future experiments. Protein concentration was determined using the BCA Protein Quantitation Kit (Beyotime Biotechnology Co., Ltd., Shanghai, China). A BCA working solution was prepared at a 50:1 ratio, and bovine serum albumin (BSA) standard solutions of varying concentrations were prepared alongside the test protein samples. Standards and samples were dispensed into a 96-well plate, followed by the addition of BCA working solution. The plate was sealed and incubated at 37 °C for 30 min to ensure a complete reaction. After incubation, the plate was cooled to room temperature, and a microplate reader was used to measure absorbance at 562 nm. A standard curve was generated from three replicates of the standards, plotting concentration against absorbance. Protein concentrations of the test samples were calculated by comparing their absorbance values to the standard curve.

### 2.7. Western Blot Analysis

TBX3 protein was separated, detected, and identified using SDS-PAGE electrophoresis and antibody incubation techniques. To ensure complete denaturation of the protein samples prior to electrophoresis and maintain the accuracy of subsequent analyses, the supernatant obtained in the previous step (Section 2.6) was boiled at 99 °C for 10 min. An SDS-PAGE gel (Solarbio Science & Technology Co., Ltd., Beijing, China) was prepared, consisting of an 8% resolving gel and a 5% stacking gel. During the electrophoresis process, the gel concentration phase was conducted at a voltage of 80 V for 18 min, followed by a separation phase at 150 V for 55 min. This setup effectively separated proteins based on their molecular weights, resulting in distinct protein bands. Following electrophoresis, the proteins were transferred to a membrane for 48 min at a current of 280 mA. To evaluate the effectiveness of the transfer, the membranes were stained with Ponceau S solution for 5 min. Unbound dye and impurities were removed with multiple 10 min washes using PBST buffer. To minimize nonspecific binding, the membranes were blocked with a 5% skim milk solution for 2 h, followed by overnight incubation at 4 °C with primary antibodies. TBX3 protein was targeted using a TBX3-specific rabbit pAb (1:700 dilution, Bioss Antibodies, Beijing, China), and β-Tubulin was used as an internal control, detected with anti-β tubulin (1:500 dilution, Abcam, Cambridge, MA, UK). After incubation for up to 15 h, unbound primary antibodies were removed by washing the membranes three times for 10 min each with PBST buffer. A secondary antibody, goat anti-rabbit IgG (H + L) HRP (1:5000 dilution, Affinity Biosciences, Changzhou, China), was added and incubated for 2 h at 37 °C on a shaker to facilitate binding to the primary antibody, forming a detectable complex. After incubation, the membrane was washed three times for 10 min each with PBST buffer to remove the unbound secondary antibody. The membranes were developed using enhanced chemiluminescence (ECL) technology (Thermo Fisher Scientiffc Inc., Waltham, MA, USA), with an exposure time of 300 s for both TBX3 and the internal control (β-tubulin).

### 2.8. Immunohistochemical Staining

Immunohistochemical staining was employed to detect and localize TBX3 protein in skin tissue samples. The sections prepared in Section 2.2 were first baked in an oven at 65 °C for 1 h to ensure optimal adhesion to the slides and minimize background staining. Following baking, the sections were placed into xylene solution twice for 15 min each time to achieve clearing, then processed with a series of alcohol gradient dehydration steps to thoroughly remove the wax from the sections. In the antigen retrieval stage, sodium citrate repair solution was used to treat the sections. This treatment exposed and restored the antigenic epitopes within the tissues, enhancing the sensitivity and specificity of the immunostaining process. The immunostaining protocol was carefully followed according to the reagent instructions. For the primary antibody titration, 60 µL of TBX3 antibody (Affinity Biosciences, Changzhou, China), diluted at a 1:200 ratio, was applied to the sections. Control sections were treated with the dilution buffer only, without the primary antibody, to assess background staining levels. The primary antibody was incubated overnight at 4 °C to allow for sufficient binding to the target antigen. The next day, the sections were removed from the refrigerator and allowed to return to room temperature. They were then rinsed three times with PBS buffer to remove any unbound antibodies and impurities. For DAB (diaminobenzidine) color development, the development time was strictly controlled at 4 min to prevent over- or under-development of the staining. Following DAB development, the sections were counterstained with hematoxylin for 3 min to highlight tissue structures. Excess stain was removed by rinsing the sections under running water, which helped enhance the clarity of the staining results. The sections were then subjected to a gradient alcohol dehydration and transparency treatment to achieve optimal clarity. Finally, the sections were sealed with neutral gum and allowed to dry naturally before being observed and photographed under a microscope for TBX3 protein localization.

The staining results were also quantitatively analyzed using WCIF ImageJ 1.37c software to measure the grayscale values of the stained sections. The data were then subjected to statistical analysis using one-way ANOVA and *t*-tests, performed with GraphPad Prism 10 software. The significance levels were set as follows: a *p*-value < 0.05 indicates a significant difference, *p*-value < 0.01 indicates a highly significant difference, and *p*-value > 0.05 indicates no significant difference (ns).

## 3. Results

### 3.1. HE Staining Results

**Figure 2:**
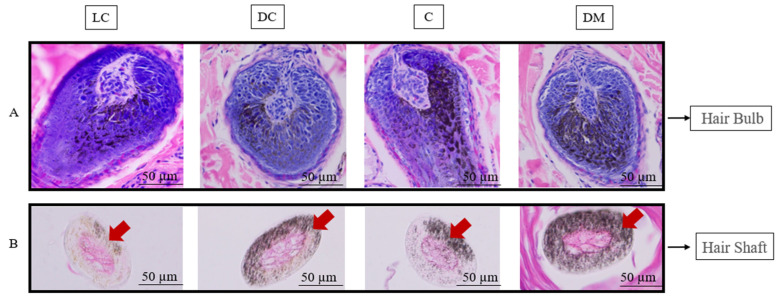
HE staining of follicle morphology and pigmentation in different parts of the dun Mongolian Bider horse. Note: LC shows the light-colored Bider markings on the shoulder; DC shows the dark-colored Bider markings on the shoulder; C shows the croup skin; and DM shows the dorsal midline. (**A**) shows the pigmentation distribution the hair bulb; (**B**) shows the pigmentation distribution in the hair shaft. Red arrow shows the location of pigment deposition. The scale used in the illustrations is 50 µm.

The HE staining results revealed a well-defined skin and hair follicle structure, including the epidermis, hair shaft, hair bulb, and distinct phases of the hair growth cycle: anagen, catagen, and telogen (Figures S2 and S3). Pigmentation distribution was specifically examined in the hair bulb and hair shaft during the anagen phase (Figure 2). Both longitudinal and transverse sections of the hair bulb and shaft demonstrated that pigmentation was most intense and uniformly distributed along the dorsal midline. In contrast, the dark-colored Bider regions exhibited a relatively symmetrical pigmentation pattern, while the light-colored Bider areas and the croup showed greater variability in pigmentation intensity and uniformity.

### 3.2. RT-qPCR Results

**Figure 3:**
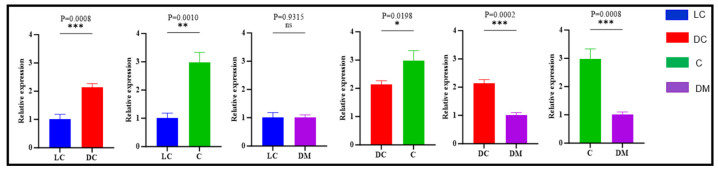
Relative expression of the *TBX3* gene in skin tissues of three different sampling sites. Note: LC shows the light-colored Bider markings on the shoulder; DC shows the dark-colored Bider markings on the shoulder; C shows the croup skin; and DM shows the dorsal midline. The error bars represent the standard error of the mean (SEM). The significance levels were set as follows: a *p*-value < 0.05 (*) indicates a significant difference, *p*-value < 0.01(**/***) indicates a highly significant difference, and *p*-value > 0.05 (ns) indicates no significant difference.

The analysis of *TBX3* mRNA expression revealed significant regional variation across different areas (Figure 3). Expression levels were significantly higher in the croup compared to the dorsal midline and in the dark-colored Bider markings compared to the light-colored Bider markings on the shoulder (*p* < 0.001). Additional comparisons indicated significant differences between the light-colored Bider markings and the croup (*p* < 0.01), as well as between the dark-colored Bider markings and the croup (*p* < 0.05). Furthermore, *TBX3* expression levels were significantly higher in the dark-colored Bider markings than in the dorsal midline (*p* < 0.01). However, no significant difference in *TBX3* expression was observed between the light-colored Bider markings and the dorsal midline (*p* > 0.05).

### 3.3. Western Blot Results

**Figure 4:**
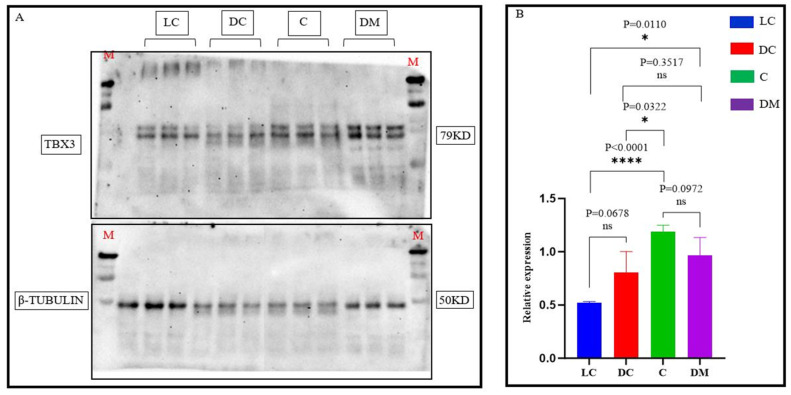
Relative expression of TBX3 protein in skin tissues from three different sites. Note: M indicates the marker; LC shows the light-colored Bider markings on the shoulder; DC shows the dark-colored Bider markings on the shoulder; C shows the croup skin; and DM shows the dorsal midline. (**A**) presents the WB (Western blot) image; (**B**) is a bar graph depicting the relative expression levels of TBX3 protein in skin tissues from the three different regions, with different colors representing different locations. The error bars indicate the standard error of the mean (SEM). The significance levels were set as follows: a *p*-value < 0.05 (*) indicates a significant difference, *p*-value < 0.01(****) indicates a highly significant difference, and *p*-value > 0.05 (ns) indicates no significant difference.

Western blot analysis revealed distinct patterns of TBX3 protein expression across the three regions (Figure 4). No significant differences in TBX3 protein levels were observed between the dark-colored and light-colored Bider markings on the shoulder or between the croup and dorsal midline (*p* > 0.05). However, TBX3 protein expression was significantly higher in the croup compared to the light-colored Bider markings (*p* < 0.0001). Additional comparisons showed significant differences between the light-colored Bider markings and the dorsal midline (*p* = 0.01), as well as between the dark-colored Bider markings and the croup (*p* < 0.05). No significant difference in TBX3 protein expression was found between the dark-colored Bider markings and the dorsal midline.

### 3.4. Protein Localization Results

**Figure 5:**
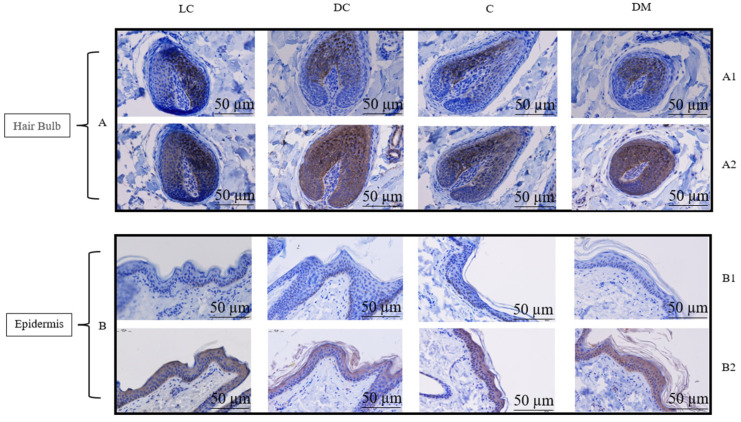
Immunohistochemical staining of different sampling locations of the dun Mongolian Bider horses. Note: (**A**). Localization of TBX3 protein in the hair bulb (A1 with primary antibody, A2 without primary antibody); (**B**). Localization of TBX3 protein in the epidermis (B1 with primary antibody, B2 without primary antibody). LC shows the light-colored Bider markings on the shoulder; DC shows the dark-colored Bider markings on the shoulder; C shows the croup skin; and DM shows the dorsal midline. The scale used in the illustrations is 50 µm.

Immunohistochemical staining results predominantly localized the TBX3 protein in the hair bulb as well as the epidermis but not in the hair shaft (Figure 5 and Figure S4).

## 4. Discussion

This study investigates the role of the *TBX3* gene in the development of Bider markings in dun Mongolian horses, offering valuable insights into its expression and localization. These findings provide a molecular basis for understanding the unique pigmentation patterns associated with this primitive marking in domestic horses. However, several factors must be considered when interpreting the results, including the small sample size and the lack of uniform coat colors among the studied individuals.

The study reveals significant regional variation in pigmentation across different anatomical sites in dun Mongolian horses with Bider markings. The dorsal midline exhibited the most intense and uniformly distributed pigmentation, while the dark-colored Bider regions displayed relatively symmetrical pigmentation patterns. In contrast, the light-colored Bider areas and the croup showed greater variability in pigmentation intensity and uniformity, which aligns with differences in sampling locations.

Interestingly, the highest levels of *TBX3* mRNA expression were detected in the croup, followed by the dark-colored Bider regions. mRNA expression was lower in the light-colored Bider areas and the dorsal midline. These findings align with previous studies suggesting a link between *TBX3* expression and the modulation of hair appearance in different body regions [6]. The observed inverse relationship between *TBX3* mRNA expression in the light- and dark-colored Bider areas suggests a complexity that may reflect differences in genetic background or color phenotypes among the sampled horses. For this study, we used one yellow dun, one red dun, and one blue dun (Grullo) as representatives of dun Mongolian Bider horses. Additionally, the expression gradient observed suggests that other genes may also influence pigmentation. For example, the *ASIP* gene has been shown to interact with *TBX3* to influence dun coat color patterns in the Polish primitive horse [8]. Immunohistochemical analysis revealed that TBX3 protein was predominantly localized in the hair bulb and epidermis, key sites of melanocyte activity that are critical for hair and skin pigmentation [11,12]. Despite the highest TBX3 protein expression in the croup, there was no significant difference between croup and the dorsal midline, which is inconsistent with mRNA expression. This discrepancy between mRNA and protein levels may suggest post-transcriptional regulation or differences in protein stability. Future studies should aim to incorporate a larger sample size and consider potential genetic and epigenetic interactions. Additionally, advances in integrative omics analyses could provide deeper insights into the regulatory mechanisms influencing *TBX3* gene expression and its role in equine pigmentation.

## 5. Conclusions

This study contributes to a deeper understanding of the molecular basis underlying Bider markings in dun Mongolian horses, emphasizing the role of *TBX3* in the regional variation of pigmentation. It also underscores the importance of investigating indigenous horse breeds to enhance our knowledge of the genetic factors driving equine coat color diversity.

## Data Availability

The original contributions presented in the study are included in the article; further inquiries can be directed to the corresponding authors.

## Supplementary Materials

The following supporting information can be downloaded at https://www.mdpi.com/article/10.3390/genes15121589/s1.

## References

[1] Linderholm A., Larson G. (2013). The role of humans in facilitating and sustaining coat colour variation in domestic animals. Semin. Cell Dev. Biol..

[2] Cieslak M., Reissmann M., Hofreiter M., Ludwig A. (2011). Colours of domestication. Biol. Rev. Camb. Philos. Soc..

[3] Librado P., Khan N., Fages A., Kusliy M.A., Suchan T., Tonasso-Calviere L., Schiavinato S., Alioglu D., Fromentier A., Perdereau A. (2021). The origins and spread of domestic horses from the Western Eurasian steppes. Nature.

[4] Ludwig A., Pruvost M., Reissmann M., Benecke N., Brockmann G.A., Castanos P., Cieslak M., Lippold S., Llorente L., Malaspinas A.S. (2009). Coat color variation at the beginning of horse domestication. Science.

[5] Raudsepp T., Finno C.J., Bellone R.R., Petersen J.L. (2019). Ten years of the horse reference genome: Insights into equine biology, domestication and population dynamics in the post-genome era. Anim. Genet..

[6] Imsland F., McGowan K., Rubin C.J., Henegar C., Sundstrom E., Berglund J., Schwochow D., Gustafson U., Imsland P., Lindblad-Toh K. (2016). Regulatory mutations in TBX3 disrupt asymmetric hair pigmentation that underlies Dun camouflage color in horses. Nat. Genet..

[7] Masuda M., Tsunoda J., Nomura H., Kimura N., Altangerel G., Namkhai B., Dolj U., Yokohama M. (2007). New Primitive Marking (Bider) in Mongolian Native Horse and Equus przewalskii. J. Equine Sci..

[8] Cieslak J., Brooks S.A., Wodas L., Mantaj W., Borowska A., Sliwowska J.H., Ziarniak K., Mackowski M. (2021). Genetic Background of the Polish Primitive Horse (Konik) Coat Color Variation-New Insight into Dun Dilution Phenotypic Effect. J. Hered..

[9] Stachurska A.M. (1999). Inheritance of primitive markings in horses. J. Anim. Breed. Genet..

[10] Lusis J.A. (1942). Striping patterns in domestic horses. Genetica.

[11] Ali S.A., Naaz I. (2018). Biochemical aspects of mammalian melanocytes and the emerging role of melanocyte stem cells in dermatological therapies. Int. J. Health Sci..

[12] Moreiras H., Seabra M.C., Barral D.C. (2021). Melanin Transfer in the Epidermis: The Pursuit of Skin Pigmentation Control Mechanisms. Int. J. Mol. Sci..

