# Supplementary material for: Expression and Analysis of TBX3 Gene in the Skin from Three Locations on Dun Mongolian Bider Horse
Source: Genes (Basel). 2024 Dec 11;15(12):1589. doi: 10.3390/genes15121589 (PMC11675668; doi:10.3390/genes15121589)
Supplement: Supplementary file 1 [file genes-15-01589-s001.zip › genes-3314252-supplementary.pdf]

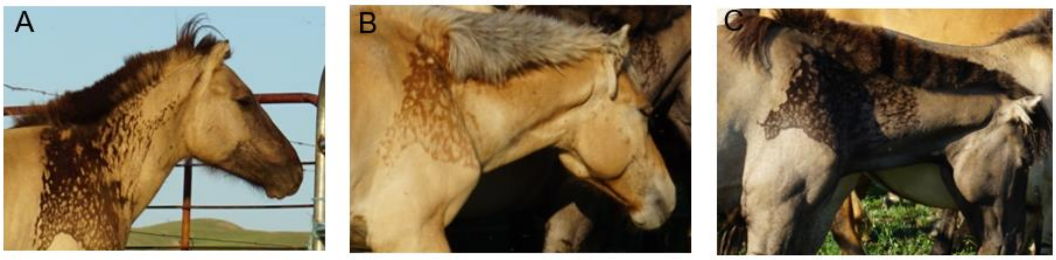

Figure S1. Dun Mongolian Bider horses with different Bider markings.

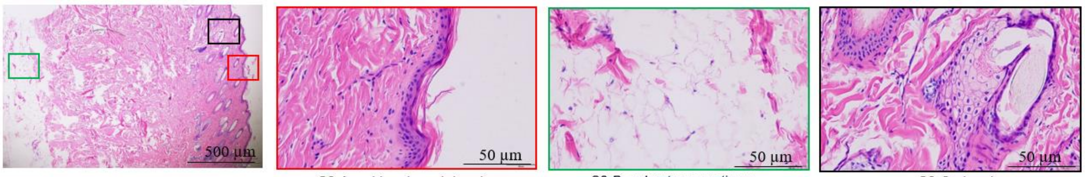

S2-A epidermis and dermis S2-B subcutaneous tissue S2-C dermis

Figure S2. HE staining reveals the skin structures.

Note: epidermis and dermis(S2-A); subcutaneous tissue(S2-B); dermis(S2-C). The scale used in the illustrations is 50 μm.

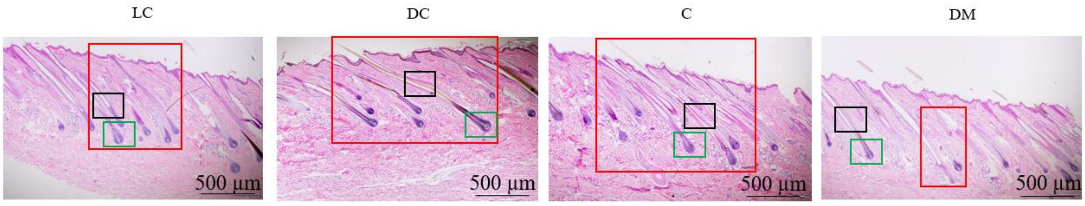

Figure S3. HE staining reveals the hair follicle growth cycles at different locations on dun Mongolian Bider horses.

Note: The red boxes highlight the hair follicle growth cycles on the skin of the shoulder, dorsal midline, and croup. Left to right: anagen, catagen, telogen, and telogen phases (red box); hair bulb(green box); hair shaft(black box). The scale used in the illustrations is 500 μm.

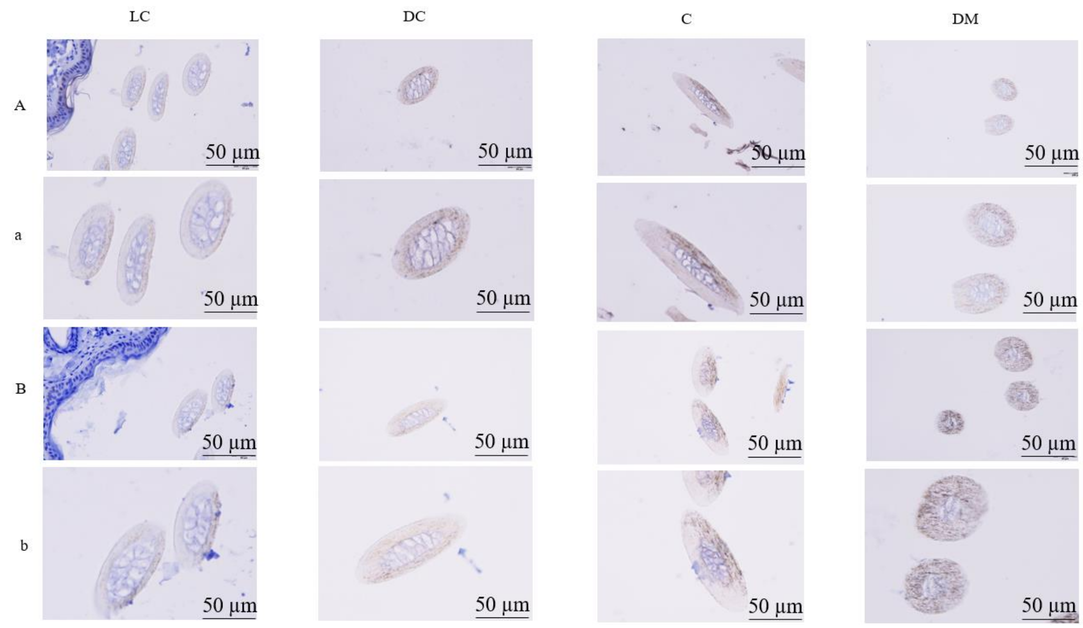

Figure S4. Immunohistochemical staining was performed on sections of hair shafts collected from the shoulder, dorsal midline, and croup.

Note: Figure A shows the positive expression of the TBX3 protein; Figure B shows the control samples. Figures a and b are enlarged images of locally cropped sections of hair shafts from different areas.

Table S1. Primer sequence information of target gene (*TBX3*) and reference gene (*B2M*)

primer name	primer sequences (5'-3')	usage	purpose
TBX3-F	CAGAGTATCCAGCGGTTGGT	target gene	RT-qPCR
TBX3-R	CACTGCAGACTGGACTGGAA		RT-qPCR
B2M-F	CTCTACTTTGGCCGCTATGTC	internal reference gene	RT-qPCR
B2M-R	CCACTTCTAAGCTGCCAGGA		RT-qPCR
